# Rocuronium for Treatment of Sugammadex Anaphylaxis: A Case Report

**DOI:** 10.1155/cria/5741904

**Published:** 2025-11-29

**Authors:** Logan H. Cone, Lauren M. Walden, Neal W. Fleming

**Affiliations:** ^1^ Department of Anesthesiology & Pain Medicine, School of Medicine, University of California, Davis, PSSB Suite 1200 4150 V St., Sacramento, 95817, California, USA, ucdavis.edu

## Abstract

Anaphylaxis presents as a complex and unpredictable intraoperative emergency. Rocuronium is among the most common triggering agents for intraoperative anaphylaxis. Sugammadex has been suggested as a therapeutic option for the treatment of anaphylaxis triggered by rocuronium. With increased use, the incidence of anaphylaxis triggered by sugammadex may approach that of rocuronium. We present a case suggesting the converse might also be efficacious. In a critically ill patient with refractory anaphylaxis apparently triggered by sugammadex, administration of rocuronium rapidly restored hemodynamic stability.

## 1. Introduction

Hypersensitivity or allergic reactions present as a complex constellation of symptoms that can be difficult to diagnose and treat in the intraoperative setting. Symptoms may be urticarial, gastrointestinal, respiratory, or cardiovascular [1]. Causation can be attributed to a multitude of items including medications such as neuromuscular blocking agents (NMBAs), antibiotics, sugammadex or protamine, and environmental exposures such as latex or chlorhexidine [2]. The most severe hypersensitivity reaction, anaphylaxis, presents as a complex and unpredictable intraoperative emergency.

The most common triggering agents for intraoperative anaphylaxis (60%) are NMBAs including succinylcholine and rocuronium [3]. Rocuronium anaphylaxis is typically treated symptomatically, with a combination of epinephrine and intravenous fluids. However, there are reports of refractory cases where sugammadex administration was used to bind the triggering rocuronium and stabilize the patient [4]. In a converse approach, we report a case of suspected anaphylaxis to sugammadex that resulted in persistent respiratory symptoms and cardiovascular collapse that finally stabilized after a novel treatment with rocuronium. In accordance with the Health Insurance Portability and Accountability Act, the patient provided written consent for publication of this case report.

## 2. Case Description

A 50‐year‐old male (123 kg, 178 cm, BMI 37) presented for elective coronary artery bypass graft (CABG) surgery. The patient had a history of ischemic cardiomyopathy secondary to multiple prior myocardial infarctions, systolic heart failure with reduced ejection fraction, paroxysmal atrial fibrillation, hypertension, Type 2 diabetes mellitus (diet controlled), hyperlipidemia, obesity, and obstructive sleep apnea, not requiring continuous positive airway pressure. Social history included active tobacco/cigarette use and prior methamphetamine abuse. Preoperative medications included atorvastatin (80 mg daily), losartan (25 mg daily), ezetimibe (10 mg daily), carvedilol (6.25 mg BID), spironolactone (25 mg daily), torsemide (5 mg daily), cariprazine (1.5 daily), isosorbide (30 mg daily), and nitroglycerin (0.4 mg sublingual PRN). He had no past surgical history and no known drug allergies.

In the preoperative staging area, the patient confirmed tobacco use the morning of surgery, last methamphetamine use 6 months prior, and intermittent episodes of chest tightness and shortness of breath with exertion. Pertinent physical exam findings included an edentulous airway and lungs clear to auscultation. Prior to transfer to the operating room (OR), peripheral venous and radial arterial catheters were placed, and midazolam (2 mg) was administered.

In the OR, standard monitors were applied (pulse oximeter, noninvasive blood pressure cuff, electrocardiogram, and quantitative EMG neuromuscular monitor (TwitchView Blink Device Company, Seattle, WA)). Preoxygenation with 100% FiO_2_ was followed by intravenous lidocaine (100 mg), fentanyl (800 μg), propofol (20 mg), and rocuronium (100 mg). An 8.0 endotracheal tube was then placed. Following induction, a right internal jugular central venous catheter/introducer (CVC) was inserted, and a Swan–Ganz catheter (CCO/SvO_2_ Edwards LifeSciences) was floated for perioperative monitoring. Intraoperative transesophageal echocardiogram examination confirmed the preoperative findings of EF 35%–40% along with mild aortic insufficiency, mild mitral regurgitation, and mild right ventricular hypertrophy. General anesthesia was maintained with inhaled sevoflurane and additional fentanyl as clinically indicated. A continuous aminocaproic acid infusion was started per institutional protocol.

After sternotomy, saphenous vein graft harvest, and left internal mammary artery dissection, the patient was given heparin (37,000 units) and transitioned to cardiopulmonary bypass. While on bypass, he underwent a 4‐vessle CABG. Prior to separation from bypass, nitroglycerin (0.2 μg/kg/min), epinephrine (0.01 μg/kg/min), and norepinephrine (0.02 μg/kg/min) infusions were started. After separation from bypass, protamine (175 mg) was given.

General anesthesia was maintained with additional fentanyl and inhaled sevoflurane. As the hemodynamic profile stabilized, the norepinephrine infusion was tapered to 0.005 μg/kg/min and a dexmedetomidine infusion (0.4 μg/kg/hr) was initiated. During chest wall skin closure, the total dose of rocuronium received to this point was 260 mg and the EMG monitor demonstrated a post‐tetanic count of 6, so the patient was given sugammadex (200 mg) as per institutional practice. Within 3 min, there was an elevation in pulmonary inspiratory pressure (PIP) with diminished tidal volumes (TVs). Within 5 min, the ventilator was unable to achieve TV greater than 25 mL. In addition, there was a decrease in SpO_2_ from 98% to 77% accompanied by marked hypotension (arterial blood pressure [ABP] mean decreased from 85 to 34 mmHg). There was a concurrent increase in pulmonary artery (PA) mean pressure from 30 to 67 mmHg and an increase in central venous pressure (CVP) from 12 to 31 mmHg.

With the onset of the hypotension, the norepinephrine infusion was increased to 0.04 μg/kg/min and the nitroglycerin infusion was paused. Intravenous bolus doses of norepinephrine (8 μg) and vasopressin (2 doses, 1 unit each) were given. Intravenous epinephrine (30 μg) was given, followed quickly by an additional 70 μg. The FiO_2_ was increased to 100%. The hemodynamic instability continued over the next 20 min with low ABP mean and elevated PAP and PIP, difficulty achieving an adequate TV and ventilator desynchrony. Additional intravenous epinephrine (1 mg) was administered. The epinephrine infusion rate was increased to 0.03 μg/kg/min. Then, calcium chloride (1 gm) and multiple doses of phenylephrine (200 μg each) were administered. The decision was then made to readminister rocuronium. At this point, the EMG monitor was demonstrating a train‐of‐four ratio of 97%–100%. A total of 100 mg was required to abolish any post‐tetanic activity. Approximately eight minutes after the onset of complete neuromuscular blockade, there was an improved, stable MAP (100 mmHg), resolution of the elevated PIP (41–27 mbar), and TV returned to baseline (22–630 mL). In addition, there was a return of the CVP and PA mean pressures to baseline. The surgical procedure was completed without further incident. The patient remained intubated, paralyzed, and sedated for transfer to the intensive care unit. Neuromuscular function was allowed to recover spontaneously while the patient remained intubated and sedated overnight with infusions of dexmedetomidine and propofol. He was extubated almost exactly 24 h after this event.

No cutaneous symptoms were observed when the surgical drapes were removed approximately 1 hour after the hemodynamic changes, but plasma tryptase measured 2 h and 40 min after administration of the sugammadex was elevated to 20.2 μg/L (normal < 11.5 μg/L). Follow‐up skin‐sensitivity testing with rocuronium, sugammadex, and rocuronium/sugammadex complex would have provided additional corroboration, but we were not able to arrange these studies. However, based on drug administration timing, subsequent symptoms, and the tryptase elevation, sugammadex was added to the patient’s medical record list of drug allergies.

## 3. Discussion

Intraoperative anaphylaxis presents as a complex emergency with increased mortality compared to the nonoperative setting [5]. We present a case of anaphylaxis presumably secondary to sugammadex administration, treated by administration of rocuronium after failed resolution with the standard treatment protocol. The incidence of sugammadex anaphylaxis is difficult to estimate due to the length of time it has been on the market relative to other medications and limited data. Initial estimates (0.3%) have decreased with experience but still vary from 0.01% to 0.004% [6]. With increased use, the incidence of anaphylactic reactions to sugammadex may increase [6]. In Japan, where it was first introduced (2008), it is estimated that sugammadex is the inciting agent for hypersensitivity reactions in 0.0029%–0.039% of all anesthetic cases [7–9]. For comparison, recent reports from New Zealand have found the incidence of rocuronium‐induced anaphylaxis to be 0.04% and that of succinylcholine to be 0.048% [10].

Reactions to sugammadex present in a clinical scenario that differs from other perioperative reactions. It is often the only drug administered prior to the onset of symptoms, in contrast to anesthetic induction when multiple potential triggering exposures occur during a short period of time. In addition, anaphylaxis due to sugammadex has rapid onset with a mean onset time of 3 min [11]. The treatment of sugammadex anaphylaxis has been recently summarized and includes standard of care treatments such as epinephrine, fluid resuscitation, and vasopressors for hypotension management [6]. In this case, the initial symptoms were respiratory, presenting as increased peak inspiratory pressures and difficulty with mechanical ventilation. Symptoms rapidly progressed to cardiovascular collapse due to the coexisting cardiac disease. Recommended treatments were utilized in this case, but there was continued hemodynamic instability, with no marked improvement until after rocuronium administration.

Most commonly, reactions to sugammadex present shortly following extubation. Succinylcholine is recommended to facilitate reintubation because of its rapid onset of action and the uncertainty of dosing requirements for rocuronium following administration of sugammadex [6]. However, in this case of sugammadex anaphylaxis, we made the decision to administer rocuronium for two reasons. The patient was still intubated, so there was no onset time urgency, and quantitative EMG monitoring of neuromuscular function was available to assure adequate dosing of rocuronium. Unexpectedly, we noted concurrent, rapid improvement and stabilization of the hemodynamics and therefore hypothesize rocuronium as a possible treatment for anaphylactic reactions to sugammadex.

Other causes for the cardiovascular collapse were considered, most notably inadequate depth of anesthesia with a Valsalva maneuver or sympathetic response increasing the right ventricular afterload and triggering a consequent cardiovascular collapse. However, there were no preceding alterations in the anesthetics administered to the patient and no changes were observed in the electroencephalographic monitors of anesthetic depth. Patient State Index values averaged 35.6 in the 5 min prior to sugammadex administration and averaged 27.6 over the 5 min beginning 5 min after sugammadex administration. Similarly, the spectral edge frequencies averaged 13.5 Hz (right) and 13.5 Hz (left) prior to sugammadex and decreased to 3.6 Hz (right) and 4.8 Hz (left) during the same time period after sugammadex.

After the clinical introduction of sugammadex, Jones and Turkstra suggested its potential utility for the treatment of allergic reactions to rocuronium [12]. This was followed by positive case reports that have been summarized [4]. The case report presented here suggests that the converse of this therapy may also be efficacious. Just as the triggering moiety of rocuronium may be masked by the combination with sugammadex, the portion of the sugammadex molecule that triggers the hypersensitivity response may be masked by the combination with rocuronium.

We believe this is the first case report to suggest the use of rocuronium to specifically treat the hypersensitivity reactions associated with sugammadex. Support for this hypothesis is provided by animal studies of inflammatory markers demonstrating smaller changes following the combination of rocuronium and sugammadex in comparison to those seen after either drug is administered alone [13]. In addition, there are case reports of patients demonstrating a cutaneous reaction to sugammadex, but no reaction to subcutaneous injection of the rocuronium/sugammadex complex [14]. We were not able to arrange follow‐up skin testing for this patient. As a note of caution, there are also case reports of patients with suspected allergic reactions to sugammadex where subsequent skin testing suggested the reaction was actually due to the sugammadex/rocuronium complex rather than to sugammadex alone [15, 16]. Although standard protocols for skin testing in patients with suspected reactions to muscle relaxants and reversal agents have been proposed [14], there is still no consensus and so variations and vagaries regarding the timing and drug concentrations used in testing protocols make these reports difficult to interpret. Consequently, the overall incidence of a reaction to the rocuronium/sugammadex complex is not possible to predict, but the possibility must be considered. Based on our experience, in the setting of a refractory anaphylaxis presumed secondary to sugammadex, therapeutic administration of rocuronium is a treatment option that could be considered. Further evaluation of this use is needed to characterize the risk/benefit balance of this therapy.

## Conflicts of Interest

Neal W. Fleming, MD, PhD, has received contracted research support from Tsumera Pharmaceuticals, Masimo, Inc., Rostrum Medical Innovations and Edwards LifeSciences, investigator‐initiated grant support from Merck Pharmaceuticals and honoraria from Edwards LifeSciences for invited presentations. The other authors declare no conflicts of interest.

## Funding

No external funding was provided for the review or preparation of this manuscript.

## Data Availability

The data that support the findings of this study are available on request from the corresponding author. The data are not publicly available due to privacy or ethical restrictions.
